# Effects of Cardiopulmonary Bypass on Mediastinal Drainage and the Use of Blood Products in the Intensive Care Unit in 60- to 80-Year-Old Patients Who Have Undergone Coronary Artery Bypass Grafting

**DOI:** 10.5935/1678-9741.20150086

**Published:** 2015

**Authors:** Fatih Aygün, Mehmet Özülkü, Murat Günday

**Affiliations:** 1 Başkent University, Konya Research and Medical Center, Turkey

**Keywords:** Coronary Artery Bypass, Cardiopulmonary Bypass, Coronary Artery Bypass, Off-Pump, Health of the Elderly, Drainage, Blood Transfusion

## Abstract

**OBJECTIVE:**

The present study consisted of patients who underwent on-pump coronary artery bypass grafting (CABG) and off-pump CABG and investigated effect of using cardiopulmonary bypass (CPB) on the amount of postoperative drainage and blood products, red blood cell (RBC), free frozen plasma (FFP) given in the intensive care unit in 60-80-year-old patients who underwent CABG.

**METHODS:**

The present study comprises a total of 174 patients who have undergone coronary artery bypass graft (off-pump or on-pump CABG) surgery in our clinic in between 2012-2015 year.

**RESULTS:**

It was observed that the amount of drainage in the first 24 postoperative hours was lower in the on-pump CABG group (Group 1) when compared to off-pump group (Group 2) (Group 1 *vs*. Group 2; 703.5±253.8 ml *vs*. 719.6±209.4 ml; *P* =0.716). However, the amount of drainage in the second 24 hours was statistically significantly lower in the off-pump CABG group (Group 1 *vs*. Group 2; 259.8±170.6 ml *vs*. 190.1±129.1 ml; *P* =0.016). With regard to the amount of overall drainage, no statistically significant difference was observed between the two groups. Group 1 needed RBC transfusion higher than Group 2 (Group 1 *vs*. Group 2; 2.2±1.3 bag *vs*. 1.2±0.9 bag; *P* <0.001).

**CONCLUSION:**

We can say that CPB influences the amount of second 24-hour drainage which indexed body surface area. In addition, CPB decreases hct, hb, thrombocyte count in ICU arrived, after 24 hours in postoperative period. Reduced thrombocyte counting effect can be appeared after 48 hours in the postoperative period of CPB.

**Table:** 

**Abbreviations, acronyms & symbols**	
	
ACT	= Activated clothing time
BMI	= Body mass index
CABG	= Coronary artery bypass grafting
COPD	= Chronic obstructive pulmonary disease
CPB	= Cardiopulmonary bypass
CVS	= Cardiovascular surgery
FFP	= Fresh frozen plasma
Hb	= Hemoglobin
Hct	= Hematocrit
ICU	= Intensive care unit
LIMA	= Left internal mammary artery
MCPB	= Mini cardiopulmonary bypass
PAD	= Peripheral arterial disease
RBC	= Red blood cell
SPSS	= Statistical Package for the Social Sciences

## INTRODUCTION

Coronary artery bypass graft (CABG) surgery is one of the most common surgical procedures worldwide; however, it can also lead to numerous complications. The conventional CABG procedure is performed with the use of a cardiopulmonary bypass (CPB) device or heart-lung machine and is known as "on-pump" CABG, whereas coronary bypass surgery performed without the use of CPB is known as "off-pump" CABG. CABG surgery has a greater level of risk when performed in elderly patients^[1]^. Hence, cardiac surgeons have to consider the disadvantages of using CPB in older patients, particularly with regard to increased drainage and other coincident effects. Ferraris & Ferraris^[2]^ identified a relationship between the greater use of blood and its products after CABG and postoperative infection, because excessive postoperative drainage can lead to infection and other complications resulting in an increased need for blood transfusions.

On-pump CABG is considered to be the gold standard, but this method has some physiological consequences, including thrombocytopenia, activation of the complement system, immune suppression and an inflammatory response that causes organ dysfunction. In addition, manipulation of the ascending aorta during cannulation (cannulation and cross-clamping) poses the ensuing risk of embolization and stroke^[3]^. Thus, drainage tubes are inserted into the mediastinum and thoracic cavity at the end of the CABG procedure to monitor leakage and bleeding throughout these regions. A large volume of drained fluid implies the need for increased use of blood products (erythrocyte suspension and fresh frozen plasma [FFP]), and can lead to unfavorable consequences.

The risk factors for transfusion after CABG include advancing age, preoperative aspirin therapy, priority of the operation, lower preoperative volume of red blood cells (RBCs), duration of the CPB, recent fibrinolytic therapy, reoperative CABG, and differences in heparin management^[4-9]^. Off-pump CABG may reduce the need for the transfusion of blood products, lead to shorter stays in the intensive care unit (ICU), and reduce the postoperative hospitalization time^[10-12]^. However, in one previous study, there was no difference in the level of bleeding, mediastinitis, and rate of mortality between off-pump and on-pump CABG patients^[13]^.

We investigated the effects of CPB on the level of postoperative drainage and the number of blood products administered in the ICU to patients between the ages of 60 and 80 years who underwent both on-pump and off-pump CABG.

## METHODS

### Clinical characteristics of the patients

A total of 174 individuals who underwent CABG surgery at our clinic between 2012 and 2015 were included in this study. Data were collected retrospectively, and approval for the study was obtained by the ethics committee of our institution. The patients were reviewed consecutively and included in the study according to predetermined criteria. Care was taken to ensure that the make-up of each group was similar.

All of the patients had their medical history taken and underwent a detailed physical examination. In the preoperative period, standard preoperative laboratory tests including a pulmonary function test (Spirobank spirometer; MIR, Waukesha, WI, USA), transthoracic echocardiography (Sequoia C256; Acuson, Mountain View, CA, USA), and bilateral carotid artery Doppler ultrasonography (Xario prime ultrasound; Toshiba, Japan) were performed in the cardiovascular surgery (CVS) clinic. Calcifications in the thoracic and ascending aorta and aortic arch were evaluated by chest radiography prior to surgery. During surgery, the starting points of the ascending aorta and aortic arch were thoroughly examined through manipulation. The procedure was changed if plaque was detected, and these patients were not included in the study.

During the preoperative period, clopidogrel (if any was being administered) was discontinued 5 days prior to surgery, and acetylsalicylic acid (if any) was discontinued 1 day prior to surgery in patients scheduled to undergo on-pump (crossclamped) CABG.

The same was done for those scheduled for off-pump (beating-heart) CABG.The blood glucose levels of patients with type 2 diabetes were regulated with insulin both before and after surgery, and were maintained below 200 mg/dL. Preoperative blood samples were collected in standard ethylenediaminetetraacetic acid vacuum tubes, and analyzed using a Cell-Dyn 3700 instrument (Abbott Laboratories, Abbott Park, IL, USA). Prothrombin time, activated partial thromboplastin time, and international normalized ratio levels were measured. The preoperative data of all study participants were within the normal ranges.

Dyslipidemia was defined as a fasting total cholesterol level in serum > 240 mg/dL, triglyceride level > 200 mg/dL, low-density lipoprotein cholesterol level > 160 mg/dL, and/or high-density lipoprotein cholesterol level < 40 mg/dL; anyone receiving active medical therapy for this condition was also considered to have it^[14]^. Cholesterol levels were measured using an enzymatic method.

Height and body weight were measured (SECA, Vogel & Halke, Hamburg, Germany) before surgery, and body mass index (BMI) was calculated. A staged approach was adopted in patients with a carotid artery stenosis of 70-100%, and was reserved for the post-CABG period. Preoperative data, including age, gender, incidence of chronic obstructive pulmonary disease (COPD), and peripheral arterial disease rate are provided in Table 1.

**Table 1:** Preoperative data according to groups.

	Group 1 (n=91)	Group 2 (n=83)	*P* values
	(On-pump CABG)	(Off-pump CABG)	
Age (±SD) (year)	68±5.2	67±4.6	0.437^T^
Gender (male)	53 (58.2%)	58 (69.9%)	0.118^P^
Smoking	34 (37.4%)	37 (44.6%)	0.357^P^
COPD	16 (17.6%)	21 (25.3%)	0.266^P^
Hypertension	82 (90.1%)	65 (78.3%)	0.037^P^
PAD	5 (5.5%)	1 (1.2%)	0.214^F^
Preoperative stroke story	10 (11%)	5 (6%)	0.288^P^
Diabetes no-diabetes	*59 (64.8%)	*49 (59%)	*0.439^P^
oral a/d	22 (24.2%)	26 (31.3%)	
parenteral a/d	10 (11%)	8 (9.6%)	
Right carotid artery			
stenosis<50%	55 (60.4%)	29 (34.9%)	
50%< stenosis≤70%	*5 (5.5%)	*5 (6%)	*0.641^P^
70%≤ stenosis<100%	*2 (2.2%)	-	
stenosis=100%	-	-	
Left carotid artery			
stenosis<50%	48 (52.7%)	30 (36.1%)	
50%< stenosis≤70%	*11 (12.1%)	*1 (1.2%)	*0.003^F^
70%≤ stenosis<100%	-	-	
stenosis=100%	*1 (1.1%)	-	
BMI	29.9±5.4	29.9±5.1	0.983^T^
Ejection Fraction	52.3±7.5	54.6±8.4	0.070^T^

T =*P* value was presented as a result Student-t test

P =*P* value was presented as a result Pearson chi-square test

F =*P* value was presented as a result Fischer's Exact tests

* =*P* value was calculated according to carotid artery stenosis <50%

CABG=coronary artery bypass grafting; BMI=body mass index; SD=standard deviation. PAD=peripheral artery disease; COPD =chronic obstructive pulmonary disease

### Surgical procedure

First, isolated CABG surgery was performed in all of the study participants by the same surgical team. Fentanyl, midazolam, and pancuronium bromide were administered for the induction of anesthesia. Then, standard median sternotomy was performed. The left internal mammary artery (LIMA) and other vascular conduits (including the saphenous vein and radial artery) were prepared. Heparin sodium (Nevparin^®^ flacon 25000 IU/5 mL) was administered at a dose of 300 IU/kg. CPB and a cross clamp, as well as standard aortic and two-stage venous cannulas, were used. A Jostra-Cobe heart-lung machine (model 043213 105; VLC 865, Sweden) was used. All of the patients received crystalloid cardioplegy during surgery and hot-shot cardioplegy at the end of surgery. While the LIMA was used in all of the cases, the right internal mammary artery was not used. The great saphenous vein and radial artery were used as grafts. A scrupulous aseptic regime was implemented in all of the surgeries. The unnecessary use of electrocautery, as well as luxury perfusion in CPB, was avoided. Sodium heparin was administered at a dose of 150 IU/kg in patients who underwent the beating-heart technique. Distal anastomoses were performed using the Octopus tissue stabilizer and Starfish heart positioner (Medtronic, Dublin, Ireland). Hematocrit (Hct) and hemoglobin (Hb) values were monitored every 20 min, beginning from the induction of anesthesia until the end of surgery. Intraoperative blood transfusion was performed when the Hct value decreased to 20%. These cases underwent full vascularization during the CABG procedure. Mediastinal and chest drainage tubes were situated subxiphoidally.

Proximal anastomoses to the aorta were performed with the assistance of a side clamp in both the on-pump and off-pump techniques. At the end of surgery, patients who underwent on-pump CABG received protamine hydrochloride (Protamin^®^ ampoule 1000 IU/1 mL) at appropriate doses for full-dose neutralization, and the activated clothing time (ACT) was maintained between 100 and 120. Patients who underwent off-pump CABG received appropriate doses of protamine hydrochloride, as before, to maintain the ACT between 140 and 150. Data related to the surgery, including choice of graft and graft number, are listed in Table 2. Table 3 provides the postoperative Hb and Hct values, thrombocyte count, and drainage on arrival in the CVS ICU, and at the first and second 24 hours periods.

**Table 2:** Operative data according to groups.

	Group 1 (n=91)	Group 2 (n=83)	*P *values
	(On-pump CABG)	(Off-pump CABG)	
Saphenous graft	91 (100%)	73 (88%)	-
Radial artery graft	56 (61.5%)	16 (19.3%)	<0.001^P^
LIMA gaft	89 (97.8%)	83 (100%)	-
Numbers of grafting	3.7±0.7	2.5±0.9	<0.001^T^
RBC (bag) (while in the operatin groom)	0.5±0.6	0.4±0.6	<0.001^T^

T =*P* value was presented as a result Student-t test

P =*P* value was presented as a result Pearson chi-square test

CABG=coronary artery bypass grafting; LIMA=left internal mammary artery; RBC=red blood cell

**Table 3:** Postoperative data according to groups

	Group 1 (n=91)	Group 2 (n=83)	*P *values
	(On-pump CABG)	(Off-pump CABG)	
Preoperative Hct	39.8±3.7	38.5±5.1	0.067^T^
Preoperative Hb	12.3±1.6	12.5±1.6	0.322^T^
Preoperative trombocyst count	259.4±80.6	260.9±77.1	0.899^T^
Hct (arrived ICU)	30.1±1.9	32±4.4	0.001^T^
Hb (arrived ICU)	10±0.8	10.9±2	0.001^T^
Trombocyst (arived ICU)	164.2±58.2	241.4±91.9	<0.001^T^
Hct (24^th^ hour in the ICU)	30.2±1.5	31.4±3.0	0.003^T^
Hb (24^th^ hour in the ICU)	10±0.5	10.4±1.0	0.005^T^
Trombocyst (24^th^ hour in the ICU)	187.9±74.7	228.8±78.2	0.001^T^
Hct (48^th^ hour in the ICU)	31.2±1.9	31.5±2.9	0.402^T^
Hb (48^th^ hour in the ICU)	10.4±0.6	10.5±1.0	0.495^T^
Trombocyst (48^th^ hour in the ICU)	184.1±63.7	207.5±75.2	0.029^T^
First 24^th^ hour drainage (ml)	704.3±253	713.8±209.8	0.788^T^
Second 24^th^ hour dranage (ml)	275.2±176.9	188.5±132.3	<0.001^T^
Total drainage count (ml)	979.6±374.7	902.4±299.1	0.133^T^
First 24^th^ hour drainage (ml)/m^2^	382.6±139.5	384.4±116.8	0.925^T^
Second 24^th^ hour drainage (ml)/m^2^	150.2±97.7	100.9±71.5	<0.001^T^
Total drainage count (ml)/m^2^	533.4±208.2	485.9±165.7	0.096^T^
FFP (bag) (used in the ICU)	2.7±1.7	2.9±1.3	0.366^T^
RBC (bag) (used in the ICU)	2.2±1.3	1.2±0.9	<0.001^T^

T =*P* value was presented as a result Student-t test

Hct=haematochryt; Hb=hemoglobin; CABG=coronary artery bypass grafting; ICU=intensive care unit; FFP=fresh frozen plasma; RBC=red blood cell

### Postoperative care

After the surgery was completed, the patients were admitted to the CVS ICU, and their Hct and Hb values were monitored at 4 hours intervals. The criterion for blood transfusion was an Hct value of 28%. During the postoperative period, 300 mg/day acetylsalicylic acid (Coraspin 300^®^) was commenced in all of the patients along with enteral nutrition to reduce the risk of post-CABG stroke. Cefazolin sodium (Cefamezin^®^-IM/IV), which is used as the standard antibiotic in our clinic, was initially administered at a dose of 1 g, 30 minutes before surgery, and then every 8 hours after surgery, and was continued for a total of 72 hours. Blood glucose regulation in diabetic patients was strictly maintained after surgery using 100 IU/mL insulin glargine (Lantus^®^ flacon), and 100 IU/mL human soluble insulin (Humulin-R^®^ flacon), and insulin infusions were administered when required. The blood glucose concentration was maintained below 200 mg/dL in all of the diabetic patients.

The patients' drainage tubes were checked over a 48 hours period after the patient was admitted to the CVS ICU. After a stay in the ICU, patients were transferred to the CVS clinic with their drains and arterial catheters removed, and on postoperative day 4, the central venous line was removed. Patients were discharged from the hospital on postoperative days 6-11.

### Statistical analyses

Statistical analyses were performed with Statistical Package for the Social Sciences (SPSS) software (SPSS Inc., Chicago, IL, USA). The statistical significance of nonparametric data between the groups was analyzed by a Chi-square test and Fisher's exact test, because the observed values were below those expected. While the parametric data were represented as minimum, maximum, and mean ± standard deviation, the statistical significance of the parametric data between the groups was analyzed by an independent Student's t-test. Results were considered statistically significant at *P* <0.05.

### Study groups

Consecutive patients who underwent the CABG procedure between 2012 and 2015 were grouped randomly according to two different surgical techniques. The first group (Group 1) consisted of patients who underwent CABG by CPB and the cross-clamp (on-pump with cross-clamp) technique. The second group (Group 2) consisted of patients who underwent CABG by the beating-heart (off-pump) technique. Proximal anastomosis was performed using side clamps. The duration of use of the cross-clamp did not exceed 90 minutes, and the length of CPB did not exceed 120 minutes in Group 1.

To create a homogeneous group, the exclusion criteria were as follows: hypoalbuminemia (a serum albumin level < 3.5 gr/dL); nutritional disorders; dialysis or a serum creatinine level of 2 gr/dL; aortic pathology detected during surgery and during which the operational procedure was changed; needing postoperative intra-aortic balloon pump support; and having undergone surgery under emergency conditions, reoperative CABG, CABG without a touching ascending aorta (no-touch), LIMA-left anterior descending (for single vascular disease patients) CABG, a second CABG, valvular and CABG during the same session, or surgical exploration for any other postoperative reason, including chronic renal insufficiency and dialysis.

## RESULTS

In all, 91 (52.2%) patients underwent CABG by CPB, and 83 (47.8%) underwent CABG by the beating-heart technique. The age range of subjects was 61-79 years (mean ± standard deviation, 68±5 years); there were 111 (63.8%) males and 63 (36.2%) females. There were 147 (84.5%) patients with hypertension, 71 (40.8%) smokers, and 37 (21.3%) patients with COPD (Table 1). The number of grafts was 3.1±1 (Table 2). The preoperative Hct value was 39.2±4.4%, the Hb was 12.4±1.6 gr/dL, and the thrombocyte count was 260.1±78.7. At the time of admission to the CVS ICU after surgery, the Hct value was 31±3.5%, the Hb was 10.4±1.5 gr/dL, and the thrombocyte count was 201.1±85.2. The drainage was 708.9±232.8 mL during the first postoperative 24 hours, whereas it was 233.9±162.7 mL during the second 24 hours. The number of RBC collection bags administered was 1.7±1.2 and the number of bags of FFP was 2.8±1.5 (Table 3).

**Fig. 1:**
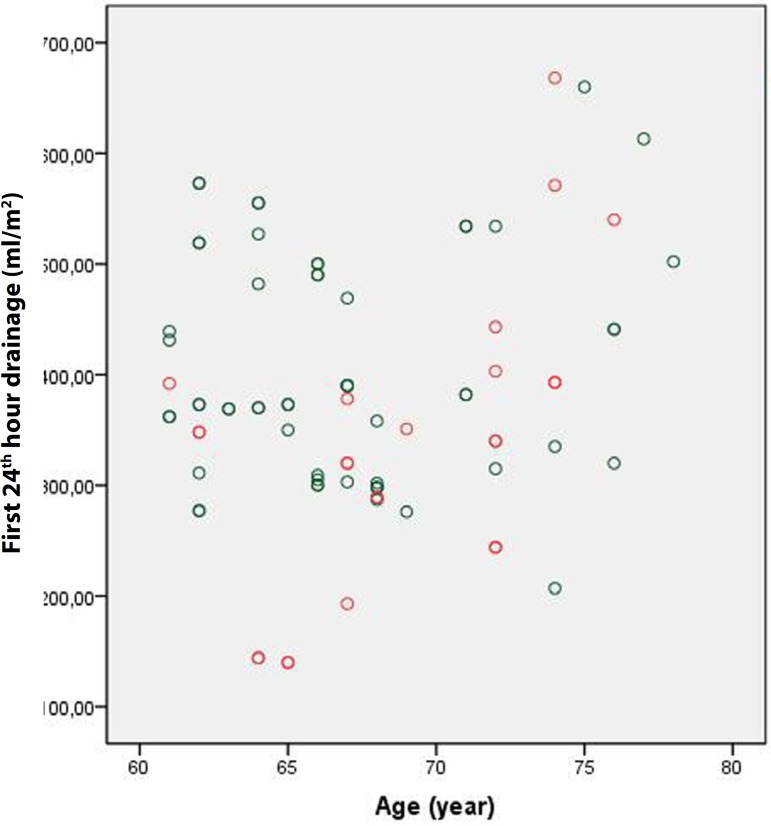
Group 1 first 24 hours drainage.

**Fig. 2:**
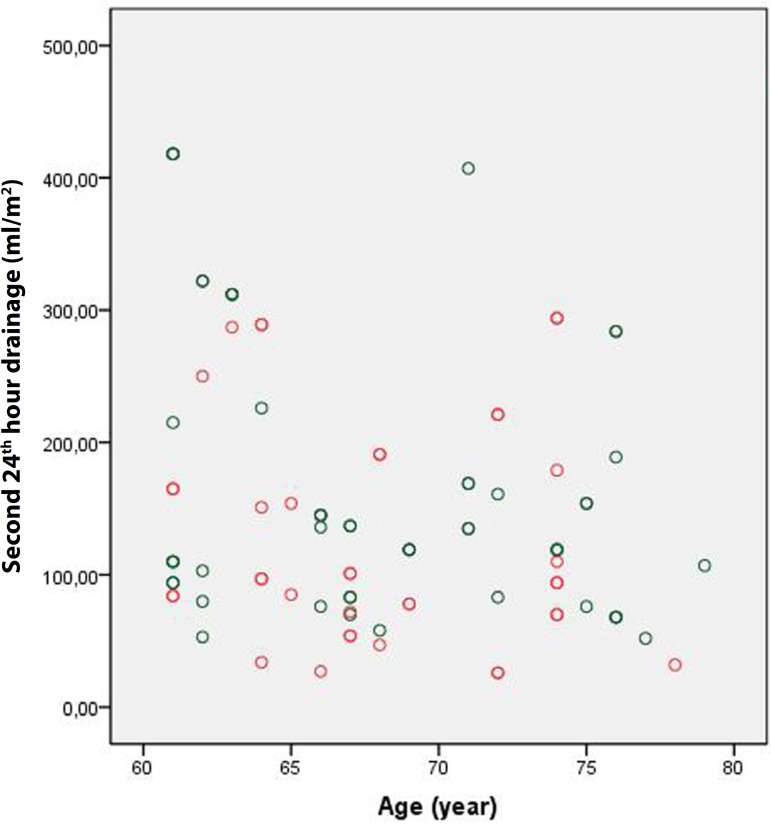
Group 2 first 24 hours drainage.

**Fig. 3:**
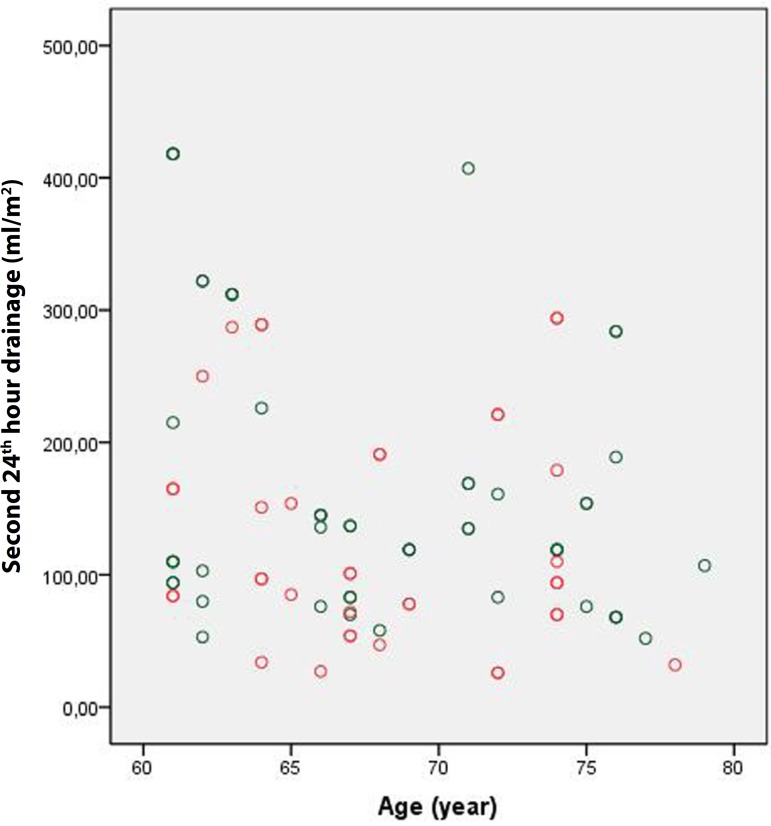
Group 1 second 24 hours drainage.

**Fig. 4:**
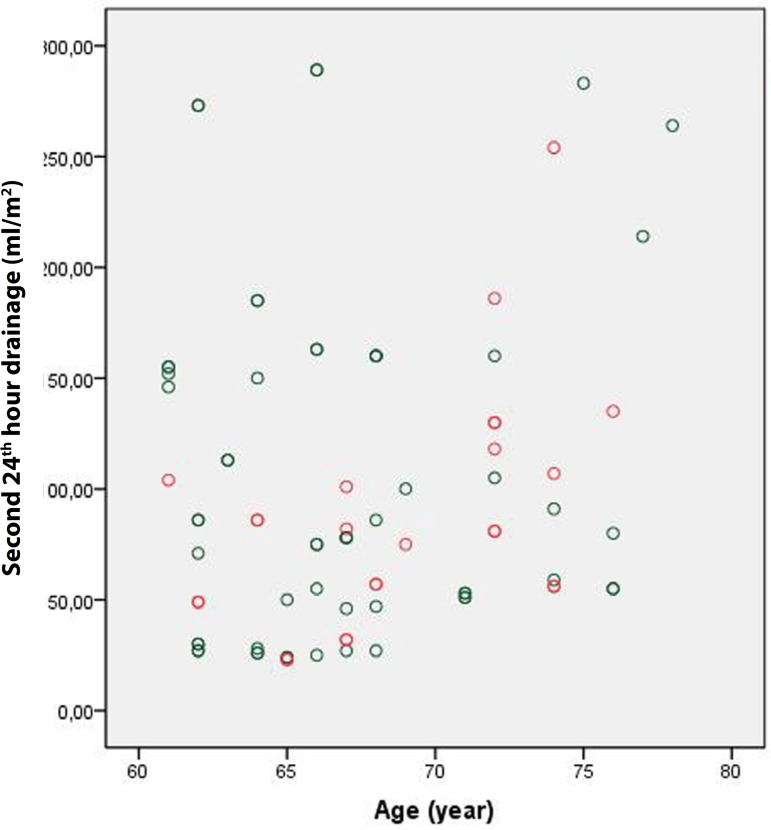
Group 2 second 24 hours drainage.

Although it was not statistically significant, the amount of drainage during the first postoperative 24 hours was lower in Group 1 than in Group 2 (704.3±253 mL *vs*. 713.8±209.8 mL, respectively; *P* =0.788). Indexing this value according to body surface area led to similar results (382.6±139.5 mL *vs*. 384.4±116.8 mL, respectively; *P* =0.925) (Figures 1 and 2). However, the amount of drainage during the second postoperative 24 hours was significantly lower in Group 1 than in Group 2 (275.2±176.9 mL *vs*. 188.5±132.3 mL, respectively; P<0.001). Again, indexing this value led to similar results (150.2±97.7 mL vs. 100.9±71.5 mL, respectively; *P* <0.001) (Figures 3 and 4).

**Fig. 5:**
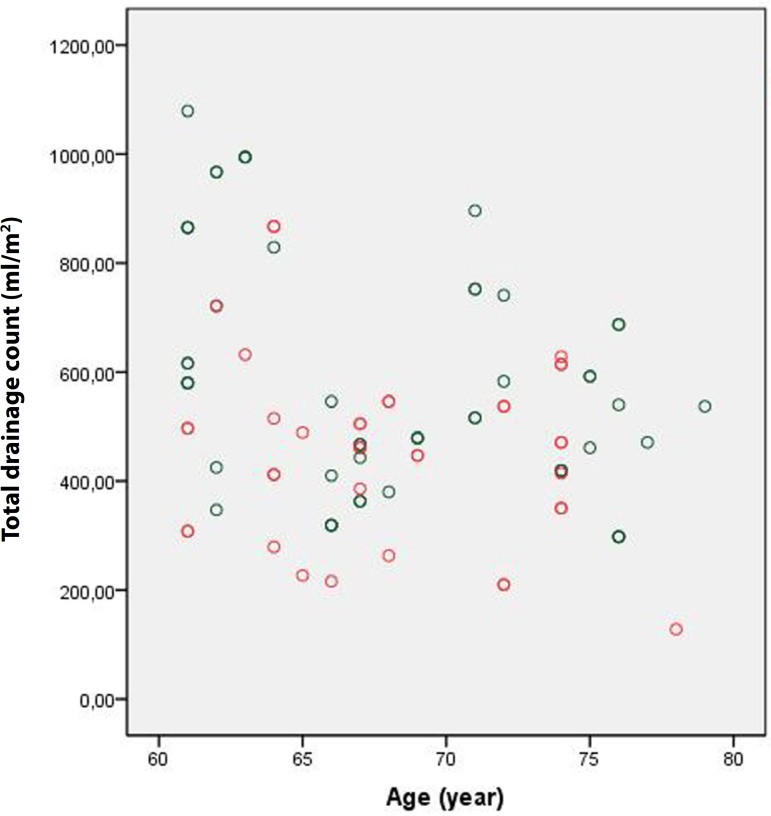
Group 1 total drainage.

**Fig. 6:**
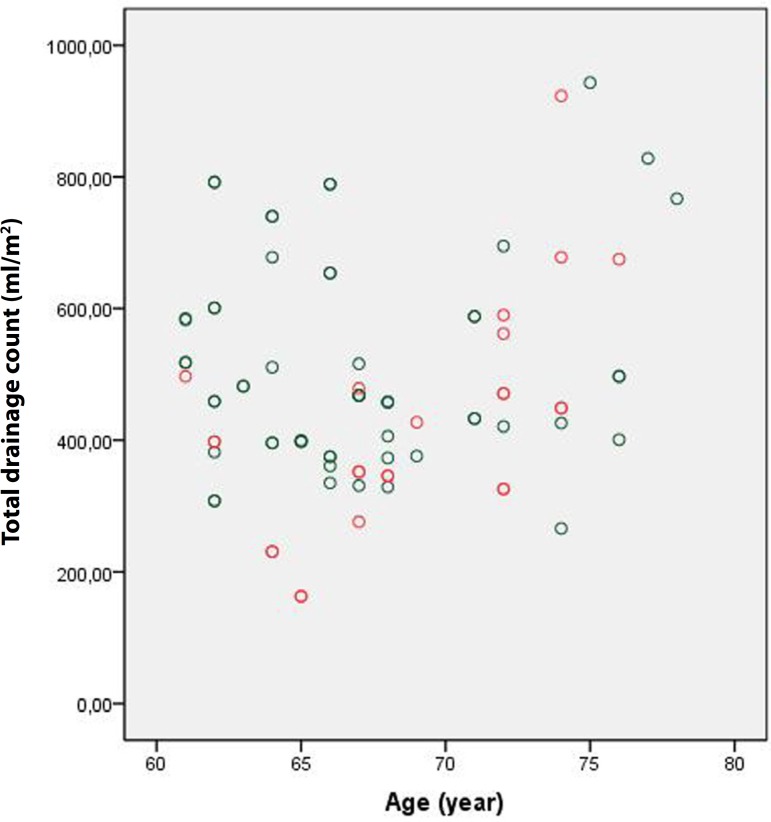
Group 2 total drainage.

With regard to the amount of overall drainage, no statistically significant differences were observed between the two groups (Figures 5 and 6). The mean number of bypass grafts used and the use of the radial artery were significantly higher in Group 1 than in Group 2. In addition, the Hct and Hb values and thrombocyte count were significantly lower in Group 1 at the time of admission to the ICU; however, the thrombocyte count subsequently recovered to the value of the off-pump group after 48 hours. In addition, there were no differences in mean Hct and Hb values between the groups at the end of 48 hours (Table 3).

## DISCUSSION

The conventional CABG procedure is characterized by precisely performed coronary anastomoses using CPB. However, ensuring a bleeding-free surgical area and performing precise coronary anastomosis using CPB may lead to complications such as blood trauma, inflammatory responses, a negative nonpulsatile flow, and potential debris embolization^[3]^. Off-pump CABG is considered to be a means of avoiding such unfavorable outcomes of CPB. Therefore, we included 91 (52%) on-pump CABG patients in our study.

Recently, CABG has been performed in a growing number of patients with a higher risk profile. The benefits of off-pump CABG with regard to aortic complications are obvious. Recent studies have demonstrated an improvement in high-risk patients who have undergone off-pump CABG^[15-18]^. In our study, we included 83 (48%) off-pump CABG patients.

Off-pump and the gold standard on-pump CABG have been compared in both large retrospective observational studies and randomized controlled studies. The results of these studies have revealed comparable outcomes. However, there is a lack of small, prospective, randomized and controlled studies in the literature. Some reports have indicated that such studies are unable to demonstrate the early and long-term outcomes related to incomplete revascularization, a decreased rate of long-term graft patency, enhanced repetitive revascularization, and survival. This has discouraged research on off-pump CABG and led to the abeyance of this technique. Other reports have claimed that studies that doubt the applicability and benefits of off-pump CABG have ignored research indicating comparable long-term outcomes and a greatly reduced period of hospitalization with the on-pump technique^[19-23]^.

Van Dijk et al.^[24]^, Angelini et al.^[25]^, and Cooley^[26]^ identifieda greater amount of drainage following on-pump CABG as opposed to off-pump CABG. Abdel Aal et al.^[27]^ identified hemodilution as the reason for the difference between the two techniques. In our study, we did not find a statistically significant difference in either the first postoperative 24 hours drainage volume or the total drainage between the two groups. The second postoperative 24 hours drainage was significantly higher in Group 1 (*P* <0.001). When we indexed the drainage according to the body surface area, we achieved similar results.

Beghi et al.^[28]^ and Hall et al.^[29]^ emphasized that, besides hemodilution, blood and air contact, hypothermia, and use of the CPB device also contribute to this difference. In our study, the Hct and Hb values, and thrombocyte count upon arrival in the CVS ICU and also after the first postoperative 24 hours, were significantly lower in Group 1 (*P* =0.01; *P* =0.01; *P* <0.01, respectively, on ICU arrival, and *P* =0.003; *P* =0.005; *P* =0.001, respectively, after 24 hours). After 48 hours, only the thrombocyte count was significantly lower (*P* =0.029).

Tsai et al.^[30]^ reported an almost similar amount of drainage in their on-pump and off-pump CABG groups, with a slightly lower volume observed in the off-pump CABG group. The authors found that a shortened CPB time and elimination of hypothermia reduced the amount of postoperative drainage during the on-pump CABG procedure. In our study, the length of time that the cross-clamp was used did not exceed 90 minutes, and the duration of CPB did not exceed 120 minutes, for patients in Group 1.

Panday et al.^[31]^ determined that the amounts of blood and blood products used in cases of off-pump CABG were slightly lower than in cases of CABG performed by the on-pump technique. In addition, the amounts used preoperatively were almost equal between a group that underwent mini cardiopulmonary bypass (MCPB) and an on-pump CPB group. We did not find a statistical significance in terms of the postoperative use of FFP between the two groups (*P* =0.036); however, RBC usage in the ICU following CABG surgery was significantly higher in Group 1 (*P* <0.001).

Ranucci & Isgrò^[32]^ reported that MCPB implementations are characterized by decreased postoperative bleeding, but not by a low transfusion rate. Folliguet et al.^[33]^ compared the postoperative transfusion rates of patients who underwent MCPB to conventional CPB, and found no statistically significant differences between the two groups. The researchers also noted the importance of the roles of anesthesiologists and pump technicians during surgery.

### Study Limitations

In the present study, all study participants were Caucasians and do not represent the other ethnic groups. Patients that would impair the similarity between the groups, such as patients with renal insufficiency and dialysis patients and redo CABG cases etc., have not been included in the study.

## CONCLUSION

The use of CPB increases the level of drainage during the second 24 hours postoperative period (Group 1 *vs*. Group 2; 275.2±176.9 mL *vs*. 188.5±132.3 mL; *P* <0.001), and also increases the second 24 hours drainage volume if indexed by the body surface area (Group 1 *vs*. Group 2; 150.2±97.7 mL/m^2^*vs*. 100.9±71.5 mL/m^2^; *P* <0.001) in 60- to 80-year-old patients who undergo the CABG procedure. We found that CPB decreased Hct and Hb values and thrombocyte counts on arrival at the ICU and after 24 postoperative hours. In addition, CPB increased RBCs usage during the intraoperative (Group 1 *vs*. Group 2; 0.5±0.6 bag *vs*. 0.4±0.6 bag; *P* <0.001) and postoperative periods (Group 1 *vs*. Group 2; 2.2±1.3 bag *vs*.1.2±0.9 bag; *P* <0.001). These data should be verified by large studies in the future.

**Table:** 

**Authors' roles & responsibilities**		
FA		Execution of surgeries and/or experiments; analysis and/ or interpretation of data; statistical analysis; writing of the manuscript or critical review of its contents; final approval of the manuscript
MO		Design and drawing of the study; final approval of the manuscript
MG		Final approval of the manuscript
